# Study of a High-Precision Read-Out Integrated Circuit for Bridge Sensors

**DOI:** 10.3390/mi14112013

**Published:** 2023-10-29

**Authors:** Xiangyu Li, Pengjun Wang, Hao Ye, Haonan He, Xiaowei Zhang

**Affiliations:** 1 College of Electrical and Electronic Engineering, Wenzhou University, Wenzhou 325035, China; lixiangyu@nbu.edu.cn (X.L.); yehao@wzu.edu.cn (H.Y.); 2 Faculty of Electrical Engineering and Computer Science, Ningbo University, Ningbo 315211, China; 2111082280@nbu.edu.cn

**Keywords:** high precision, read-out circuits, sigma-delta modulator, digital filter

## Abstract

Bridge sensors are widely used in military and civilian fields, and their demand gradually increases each year. Digital sensors are widely used in the military and civilian fields. High-precision and low-power analog-to-digital converters (ADCs) as sensor read-out circuits are a research hotspot. Sigma-delta ADC circuits based on switched-capacitor topology have the advantages of high signal-to-noise ratio (SNR), good linearity, and better compatibility with CMOS processes. In this work, a fourth-order feed-forward sigma-delta modulator and a digital decimation filter are designed and implemented with a correlated double sampling technique (CDS) to suppress pre-integrator low-frequency noise. This work used an active pre-compensator circuit for deep phase compensation to improve the system’s stability in the sigma-delta modulator. The modulator’s local feedback factor is designed to be adjustable off-chip to eliminate the effect of process errors. A three-stage cascade structure was chosen for the post-stage digital filter, significantly reducing the number of operations and the required memory cells in the digital circuit. Finally, the layout design and engineering circuit were fabricated by a standard 0.35 μm CMOS process from Shanghai Hua Hong with a chip area of 9 mm^2^. At a 5 V voltage supply and sampling frequency of 6.144 MHz, the modulator power consumption is 13 mW, the maximum input signal amplitude is −3 dBFs, the 1 Hz dynamic range is about 118 dB, the modulator signal-to-noise ratio can reach 110.5 dB when the signal bandwidth is 24 kHz, the practical bit is about 18.05 bits, and the harmonic distortion is about −113 dB, which meets the design requirements. The output bit stream is 24 bits.

## 1. Introduction

MEMS sensors are ubiquitous in our lives and indispensable in many applications, including process control, weighing scales, environmental monitoring, and temperature measurement [1,2,3,4]. They can be found in wafer steppers, weighing scales, mobile phones, automobiles, etc. While these sensors convert the physical signals into an electrical domain, their output voltage is small, at the millivolt level, such as thermocouples and bridge transducers (thermistor bridges, Hall sensors, and load cells) [5,6,7,8,9]. Therefore, they need amplifiers to boost such signals to levels compatible with typical analog-to-digital converter (ADC) input ranges. To achieve a sufficient signal-to-noise ratio, the input-referred error of the amplifier should be reduced to a low enough level, which means the amplifier must have low thermal and 1/*f* noise, high accuracy, and low drift [10,11]. Achieving all these is quite challenging in today’s mainstream CMOS technology, whose inherent precision is limited by 1/*f* noise, component mismatch, gain error, and drift. A further challenge is to achieve good power efficiency since many sensor systems are battery-powered [12,13,14,15]. This is also essential for precision temperature measurement to restrict local self-heating errors. 

With the continuous development of electronic science and technology since humankind entered the electronic information age, whether, in the defense or civilian context, the demand for electronic products is getting higher and higher, especially in the portable consumer electronics market, which is rising in the general situation. The renewal of electronic products and the rapid development of mixed-signal systems are increasingly demanding [16,17,18]. The sensor can be regarded as a device that forms the interface between non-electrical physical domains and the electrical domain. High-precision analog-to-digital converters (ADC) for Wheatstone bridge sensors have a significant market share in the corresponding field [19]. The ADC circuit architecture includes Flash, Pipeline, SAR, and Sigma-delta. Sigma-delta ADC requires lower process matching, pre-stage anti-alias filter performance, and higher conversion accuracy than other ADCs [20,21]. It uses oversampling and noise-shaping techniques to achieve high-precision analog-to-digital conversion. The analog interface circuit has a small bandwidth and high output signal accuracy, which is suitable for sigma-delta ADCs to achieve low-speed, high-precision signal conversion. In addition, the sigma-delta architecture can also be used to achieve low-power ADCs for biomedical and wireless communication systems, such as always-on chips and image sensors [22,23,24]. 

The sigma-delta modulation technique is widely used in analog-to-digital converters to convert analog signals to high-precision digital signals, generally used in applications requiring high accuracy but low speed [25]. The sigma-delta modulator is a crucial part of determining the ADCs conversion accuracy and affecting the entire system’s performance [26]. Previous researchers designed a discrete-time band-stop filter in the read-out circuit to eliminate the offset ripple [27]. However, the discrete-time switched capacitor filter will introduce noise aliasing and improve the noise power spectral density of the baseband. At the same time, this topology requires high chopping frequencies and ensures sufficient phase margin. This work proposes that the sigma-delta modulator can realize noise shaping effectively at a high oversampling ratio (OSR) with moderate speed, avoid noise aliasing, and increase power consumption. The sigma-delta circuit implemented with switched-capacitor topology has the advantages of a high signal-to-noise ratio, a large bandwidth, good linearity, and better compatibility with CMOS processes. 

For a magnetoresistive sensor, we need to accurately measure the changing magnetic signal. The signal bandwidth allows us to capture these high-frequency variations in the signal, ensuring the integrity of our measurements. The bandwidth helps filter out high-frequency noise and interference that may be present in the environment. This ensures that the acquired data are of high quality and not corrupted by external factors. This work designs and implements a fourth-order single-loop fully feedforward sigma-delta modulator and digital extraction filter that meets an accuracy of 24-bit and achieves a signal bandwidth of 24 kHz. The modulator circuit consists of a low distortion gain bootstrap op-amp, a CMOS switch, a dynamic comparator and feedforward summing circuit, an associated dual sample and hold circuit, and an active pre-compensator. The digital filter with a three-stage cascade structure consists of the following: CIC filter, CIC compensation filter, and FIR low-pass filter, and the system modeling and simulation are verified in MATLAB for all levels of filters. The layout design and engineering circuit were fabricated by a standard 0.35 μm CMOS process from Shanghai Hua Hong, and the performance of the sigma-delta ADC was tested. 

## 2. Sigma-Delta Modulator

In this work, the noise analysis of model theory is conducted for the first-stage integrator, system pre-stage noise, continuous-time filters, and switched-capacitor filter cascade characteristic variation. In order to derive the limited optimization conditions between the integrator’s pre-stage noise, quantization noise, system stability, and other parameters, we analyzed the total circuit noise of the sigma-delta modulator. 

The target of this work is a bandwidth of 24 kHz and an output bitstream of 24 bits. The relationship between the maximum signal-to-noise ratio of the system and the order (L), the oversampling rate (OSR), and the number of quantization bits (N) can be obtained using the Matlab program [28]. The relationship is shown in Equation (1):

(1) $$\begin{array}{l} SNR_{\max}=10\mathrm{lg}(\frac{P_{s}}{P_{n}})=10\mathrm{lg}(\frac{3\times 2^{2N}\times (2L+1)\times OSR^{3}}{2\pi^{2L}})\\ =6.02N+(2L+1)\times 10\mathrm{lg}OSR+1.76+10\mathrm{lg}(2L+1)-9.94L \end{array}$$

**Figure 1:**
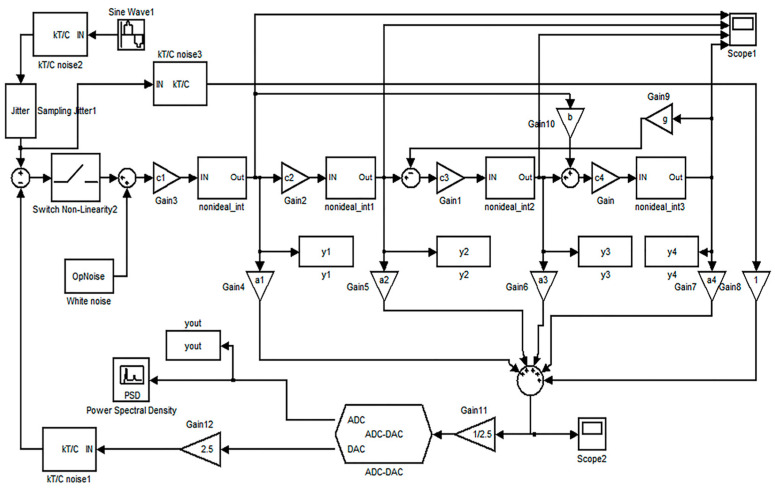
Modulator Simulink is a non-ideal model.

According to the requirements of the system specifications, we considered system stability, power consumption, complexity, etc., and the modulator structure is finally selected as a complete feedforward structure with the parameters set to OSR = 128, L = 4, and *n* = 3, which leaves a considerable design margin in this work. The system model of the modulator designed in this work is shown in Figure 1. Compared with the traditional fourth-stage feedforward structure, this structure uses local negative feedback and negative feedforward for optimizing the zero pole of the system, thus improving the system’s stability. A feedforward path is introduced at the output of the first stage. At the input of the modulator, the double sampling technique is used to double the equivalent sampling frequency and the signal bandwidth without increasing the circuit’s power consumption. The modulator uses a 3-bit quantization structure, so the fully differential topology reduces the nonlinearity introduced by the mismatch between the feedback capacitors. The charge amplifier is used in the local feedback, which can save chip area. The chopper technique is used in the first stage to reduce the 1/*f* noise of the modulator. The conventional analog adder is improved to calculate the quantized voltage value. In order to calculate the quantized voltage value, the conventional analog adder is modified to reduce the voltage difference between the two summations, thus reducing the swing rate. Modeling and simulating the behavior of a modulator can examine system stability, noise-shaping capability, and adaptability to parameter errors, guiding parameter optimization and circuit design. The diagram below depicts a behavioral-level model of a ΣΔ micro-accelerometer with non-ideal characteristics, including models for clock jitter, kT/C noise, operational amplifier thermal noise, limited bandwidth in the integrator’s operational amplifier, finite gain, and slew rate, among others. These non-ideal characteristic models can be installed and invoked in the Matlab Simulink toolbox or customized. Parameters within the modules must be reasonably optimized based on theoretical calculations and iterative simulations to ensure that the behavioral-level model closely simulates the performance of the actual circuit design. 

**Figure 2:**
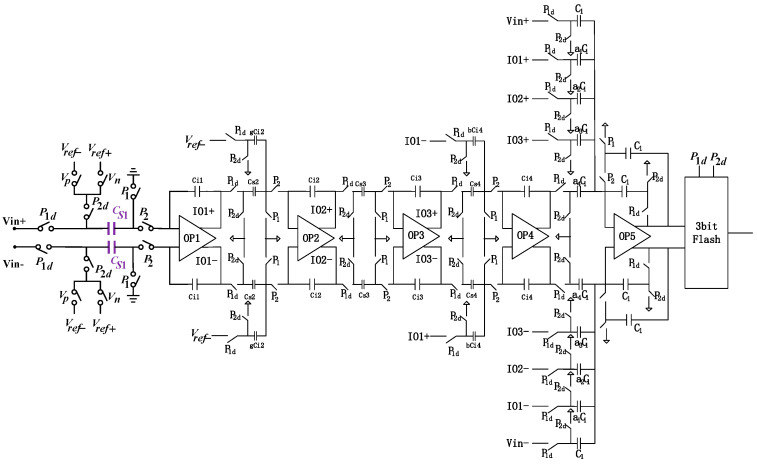
The overall circuit diagram of the Sigma-delta modulator.

We designed the modulator’s structure as a 4th-order architecture with local negative feedback as shown in Figure 2. Additionally, to ensure the system’s stability, a negative feedforward branch is extracted from the output of the first stage. An essential module within this structure is the feedback DAC unit, which is primarily responsible for implementing negative feedback and digital-to-analog conversion functions. Since it is positioned in the first stage, both noise and distortion errors will not undergo any shaping but will be directly injected into the output.

Using the sigma-delta toolbox, the values of the coefficients can be obtained, where the selection of the integration gain *c* is crucial. The significant value of *c* is better for noise shaping but unsuitable for operational amplifier implementation; however, a smaller value of *c* will result in a more significant integral capacitance, thus increasing the area. Considering the implementation of the coefficients, the system parameters are chosen as simply as possible as fractions or integers. Through optimization, the system’s parameters are shown in Table 1.

This work implements a 4th-order sigma-delta modulator using a fully differential switched-capacitor circuit. Compared with the single-end circuit, the number of capacitors and switches in the fully differential circuit is doubled. If the sampling capacitor is the same, the switch-on-resistance noise energy rises by 3 dB. However, the corresponding reference voltage is doubled at the input signal range, and the signal energy increases by 6 dB. The circuit’s total signal-to-noise ratio (SNR) still increases by 3 dB. In addition, it is known that the fully differential circuit is beneficial in improving the conversion accuracy of the modulator. At the input of the modulator, the input signal is double-sampled to increase the equivalent sampling frequency, which can double the original frequency without increasing the power consumption of the circuit, and the signal bandwidth can also be doubled as the original, which also reduces the requirements of the bandwidth and swing rate of the transconductance operational amplifier for the high sampling frequency. The separation of the input signal sampling capacitor and the feedback signal sampling capacitor in the circuit reduces the amount of charge transfer in the integration stage, improves the integrator build-up accuracy, and reduces the harmonic distortion. 

**Figure 3:**
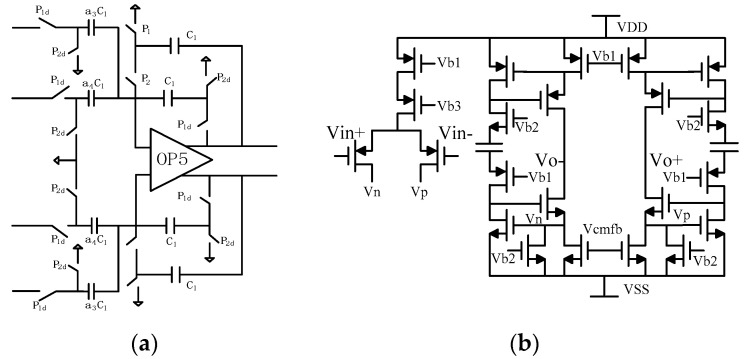
(**a**) Improved analog adder (**b**) Gain bootstrap operational amplifier.

The improved analog adder and gain bootstrap operational amplifier are as shown in Figure 3. Unlike a single-bit quantizer, where the input only needs to ensure correct polarity, multi-bit quantizers require the calculation of the precise voltage values being quantized to generate accurate feedback voltages. In P2, when it is in a high state, the output is not cleared but held. The advantage of this approach is that it reduces the voltage difference between the two summations, thereby decreasing the demands on the operational amplifier’s slew rate. 

From the overall circuit diagram, it can be observed that there are several signal input terms in the summation branch. Therefore, when designing the summing operational amplifier, the requirements for its output voltage swing need to be considered.

From the system-level modeling and simulation, it can be observed that the output signal amplitudes of the integrators and capacitor amplifiers are quite small. Therefore, a first-order folded-cascode common-source/common-gate structure can be employed for the operational amplifiers within these modules. This structure offers higher gain, a wide input common-mode range, moderate bandwidth, and relatively lower power consumption compared to second-order operational amplifiers. However, it has the drawback of a smaller output range, which is acceptable since the required range for the system is also relatively small.

On the other hand, for the operational amplifiers used in the analog adder, a second-order operational amplifier structure is utilized due to the need for a larger output range. The first-stage operational amplifier employs the structure shown in the diagram, with common-mode feedback circuitry utilizing transistor amplification. This type of common-mode feedback does not affect the gain of the operational amplifier but imposes significant limitations on the output signal range. However, due to the small output voltage swing of the integrator, this drawback can be disregarded. Through simulation, the designed operational amplifier exhibits the following characteristics: A (open-loop gain) of approximately 114.5 dB, GBWop (gain-bandwidth product) of 82.72 MHz, PM (phase margin) of 83.85°, SR (slew rate) of 66 V/μs, and thermal noise of approximately $8\;\mathrm{nV}/\sqrt{\mathrm{Hz}}$.

The equivalent load on the operational amplifier in the integration stage is approximately the sampling capacitor of that stage, while during the sampling stage, the equivalent load is approximately the sum of the sampling capacitor of the next stage and the feedforward summing capacitor. To ensure the stability of the operational amplifier, it is desirable to make the equivalent loads during the two phases as similar as possible. Based on this, the capacitance values within each integration stage and the equivalent loads on the operational amplifiers are listed in Table 2.

**Figure 4:**
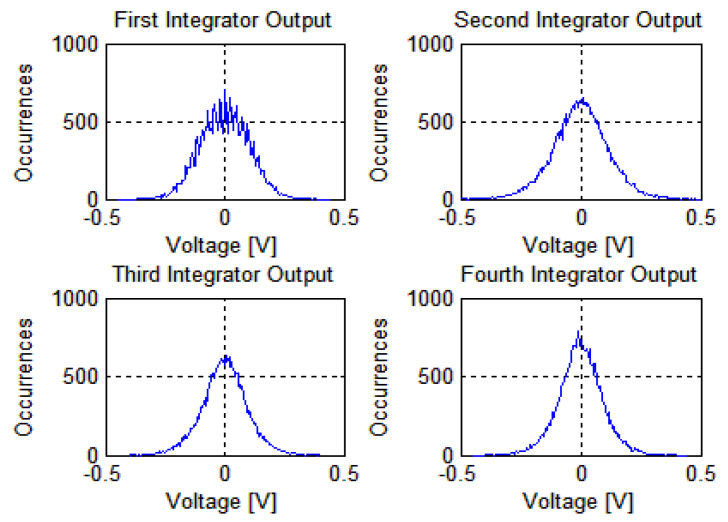
Integrator output waveform.

The overall circuit of the modulator is simulated with a clock frequency of 6.144 MHz, an input signal frequency of 25 kHz with an amplitude of −8 dBFs, and the transient simulation output waveforms of the integrators at each level are shown in Figure 4. Combining Figure 4, it can be observed that the output amplitude of the first-stage integrator is within ±0.4 V, the output amplitude of the second-stage integrator is within ±0.8 V, and the output amplitude of the third-stage integrator is within ±0.7 V. It can be seen from the figure that the output of each level integrator is stable, and the output swing is small.

**Figure 5:**
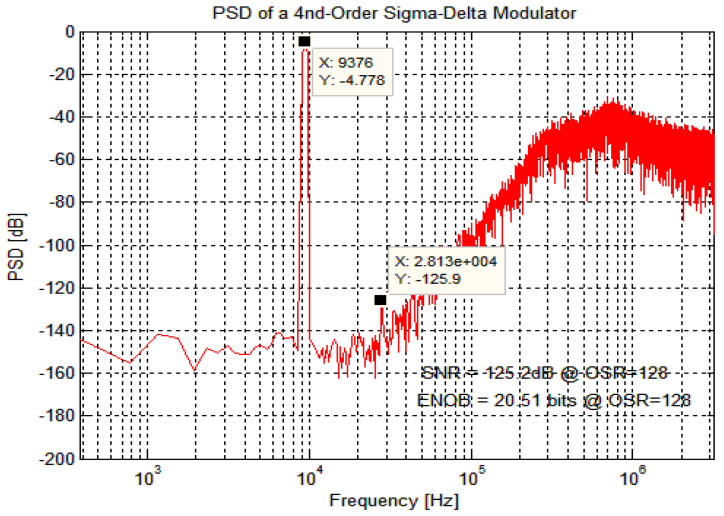
Quantifier output PSD results.

In the simulation environment of Cadence spectreVerilog, the quantizer’s output results are sampled at equal intervals, 65,536 points are captured for FFT analysis, and the PSD plot obtained by calculation and processing in Matlab is shown in Figure 5. The results show that the system realizes the noise shaping function, and the quantization noise at low frequencies is shaped to high frequencies. The signal bandwidth’s output noise level is about −140 dBV/Hz^1/2^. According to the reference voltage of ±2.5 V, the output noise voltage density in the signal band is 250 nV/Hz^1/2^. The signal-to-noise ratio can reach 125.2 dB, the effective bit count is about 20.51 bits, and the harmonic distortion is about −121 dB, which meets the requirements. 

## 3. Digital Extraction Filters

After the pre-stage sigma-delta modulator has oversampled the signal and shaped the noise, the post-stage needs to down-sample and filter the signal. The digital filter mainly performs the following functions.

1. Decimation extraction: Decimation of digital signals is used to reduce the rate to Nyquist frequency for improving the signal’s accuracy and increasing the data’s resolution.
2. Anti-aliasing low-pass filtering: Extraction is a resampling process that requires the prevention of aliasing, so anti-aliasing low-pass filtering is applied in signal processing.
3. Low-pass filtering: The quantization noise is filtered out at high frequency, expressed in the time domain as smoothing the signal waveform and increasing the SNR of the sigma-delta ADC converter.

**Figure 6:**
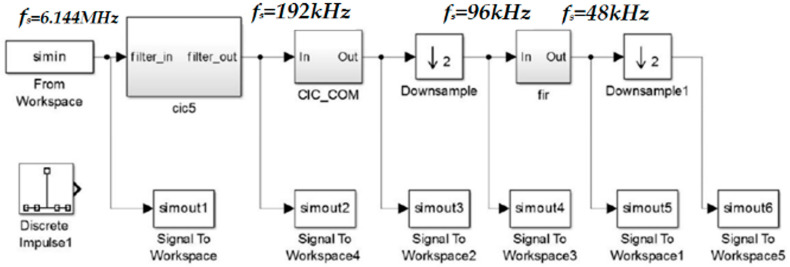
The model of the digital extraction filter.

Decimation can be seen as extracting the data stream at equal intervals from the time domain and restoring the initial signal based on the extracted data. From the frequency domain, the signal is extended with smaller frequency intervals, but if the sampling rate does not meet the requirements of Nyquist’s theorem, the signal will be mixed. If the output data rate are much higher than the Nyquist frequency, it will increase the computational burden of the system accordingly. The modulator outputs a 1-bit data stream with a large amount of data. A decimation filter is applied to reduce the sampling frequency to the Nyquist frequency and convert the high-speed unit digital signal from the modulator into a low-speed multi-bit digital signal. Usually, the back end of the sigma-delta modulator employs decimation and low-pass filtering using the CIC filter (Cascaded Integral Comb Filter) cascade scheme. The cascaded behavioral model of the back-end digital decimation filter in sigma-delta ADC is shown in Figure 6 from Simulink.

**Figure 7:**
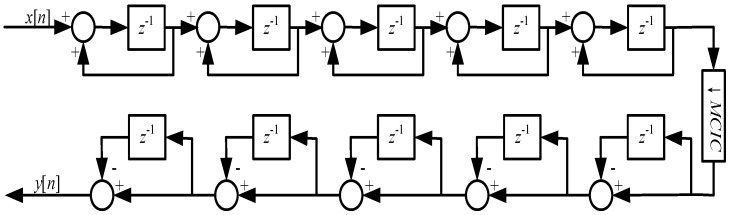
First-stage CIC filter structure.

(1) First-stage CIC filter: The CIC filter designed in this work uses a “5-stage integrator-extractor-5-stage differential” topology, which is shown in Figure 7. This topology can modify the extraction factor MCIC to control the sampling frequency and passband cut-off frequency of the overall filter, and the structure can make full use of the integrator and differential of each stage.

(2) Second stage CIC compensation filter: Since the first stage CIC has a large drop at the edge of the required passband, this results in a less than ideal passband. The second-stage CIC compensation filter flattens the passband amplitude of the CIC. According to the extraction factor MCIC = 32, the second-stage CIC compensation filter can be applied to the case where MCIC is equal to other values, and the overall passband amplitude response will always remain flat after cascading. 

(3) Third-stage FIR half-band filter: To further suppress high-frequency noise, a FIR low-pass filter is added after the second stage. The stopband attenuation of the FIR low-pass filter does not need to be large to make the aliasing small. The transition band of the half-band filter amplitude response is symmetrical about *f*_s_/4, which is effective in avoiding aliasing. The third-stage FIR low-pass filter does not need a large attenuation of the blocking band to achieve a low-aliasing extraction filtering operation. In addition, in order to further suppress the high-frequency noise, the FIR filter should have a narrow transition band. The *f*_s_ of the FIR half-band filter is set to 48 kHz, the passband cutoff frequency *f*_pass_ is 22 kHz, the stopband cutoff frequency *f*_stop_ is 26 kHz, and the passband attenuation A_pass_ is 0.001 dB for the amplitude response of the FIR half-band filter. 

The second-stage CIC compensation filter as shown in Table 3. designed based on the decimation factor MCIC = 32 can be applied without modification to other values of MCIC, and when cascaded, the overall passband magnitude response will always remain flat. The specifications of the CIC compensation filter, after parameter fine-tuning, are as shown in Table 4. To further suppress high-frequency noise, a second-stage FIR low-pass filter has been added after the second stage. As analyzed below, it is not necessary for the stopband attenuation of this stage FIR low-pass filter to be very large to keep aliasing small. The specifications of the third-stage FIR filter after parameter fine-tuning are listed in Table 4.

**Figure 8:**
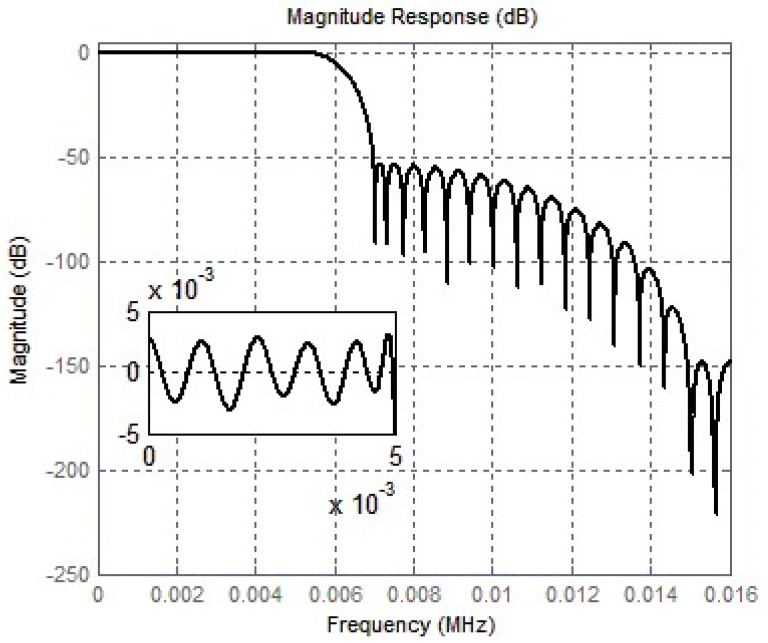
Amplitude-frequency characteristics of digital decimation filters.

The entire digital decimation filter was designed using Verilog hardware description language in Modelsim, and simulation waveform results were provided. The simulation results of the sigma-delta ADC system under Simulink were used, and the output of the modulator was used as the input signal of the decimation filter. The digital filter did not have a deteriorating effect on the performance of the entire ADC system. Then, the digital decimation filter was implemented using Verilog code in Modelsim. The model was established under Simulink, and the amplitude-frequency characteristics of the digital filter system are shown in Figure 8. The signal bandwidth reached 36 kHz, and the stopband attenuation reached −71 dB. The results met the design requirements, and it can be concluded that the designed digital decimation filter achieved 128 times the downsampling function.

## 4. Test Results and Analysis

**Figure 9:**
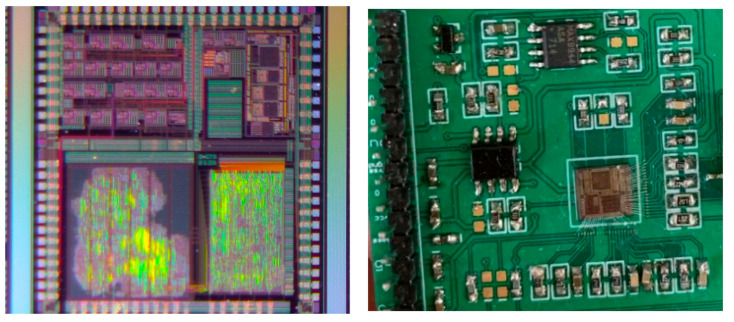
The diagram of the chip photo and the testing PCB.

With optimization improvement of circuit simulation and layout design, this work was simulated and designed in Cadence and by a four-layer metal double polycrystalline process from Shanghai Hua Hong standard 0.35 μm CMOS. The chip photo and testing PCB are shown in Figure 9. The effective area of the chip is 9 mm^2^. In order to thoroughly test the performance of the sigma-delta ADC chip, the testing PCB needs to be designed to perform detailed static and dynamic performance tests on it. An Agilent E3631 (Agilent Technologies Inc., Santa Clara, CA, USA) powers the system with a supply voltage range of 5 V to 7 V. Reducing the supply voltage reduces the overall power consumption. However, a high supply voltage helps reduce the noise of the pre-stage conversion and improves the accuracy of the output. For testing dynamic characteristics such as harmonic distortion, we used a 5 V supply, corresponding to reference voltages of 2.5 V. The Tektronix AFG3102 (Tek Technology Co., Shanghai, China) function signal generator can provide the clock signal for the modulator. Agilent 35670 (Agilent Technologies Inc., Santa Clara, CA, USA) is used to generate analog sine signals. Agilent 16804A (Agilent Technologies Inc., Santa Clara, CA, USA) is used to capture the digital signal of the modulator, and then we use the Matlab program (R2016a, MathWorks, Natick, MA, USA) to analyze the PSD of the output signal. 

**Figure 10:**
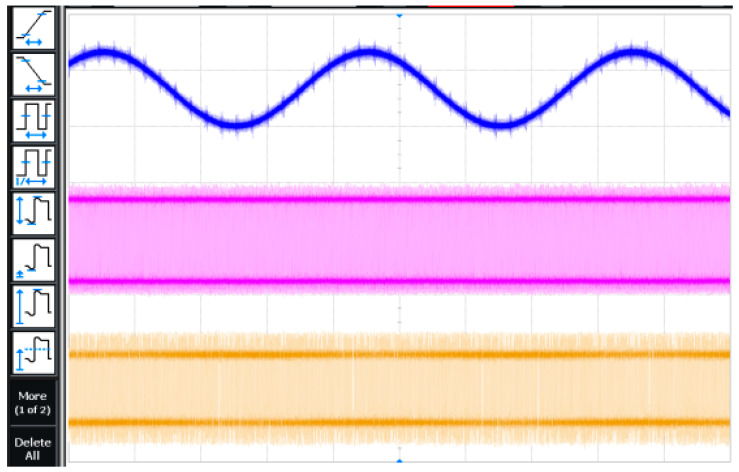
Test result of modulator transient.

**Figure 11:**
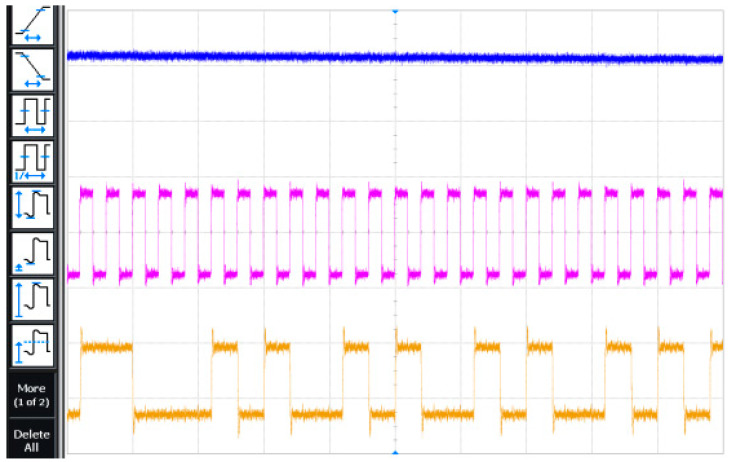
Test result of local amplification.

The transient simulation result of the modulator is shown in Figure 9 when the sampling frequency of the modulator is 6.144 MHz, the input sinusoidal signal frequency is 24 kHz, and the amplitude is −6 dBFs. The waveform in Figure 10 is the input signal, the clock signal, and the digital output signal in order. Figure 11 is the local amplification result of Figure 10. The brief graphical comparison shows that it can achieve the function of analog-to-digital conversion. 

**Figure 12:**
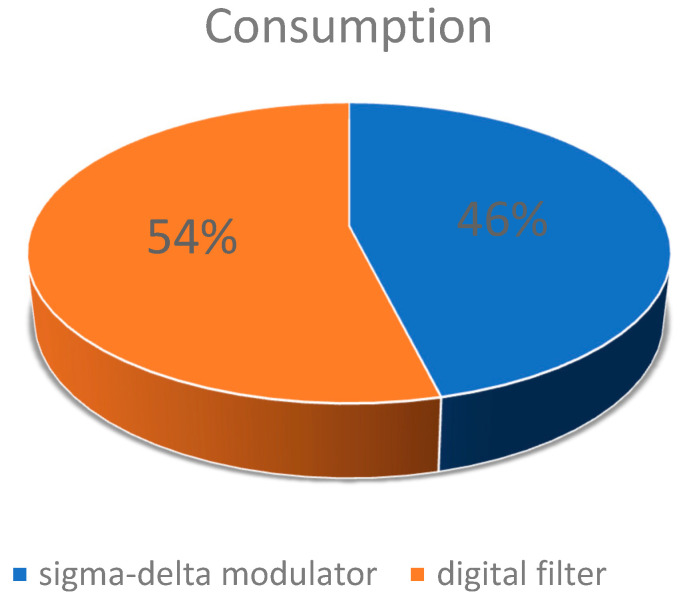
Power consumption.

**Figure 13:**
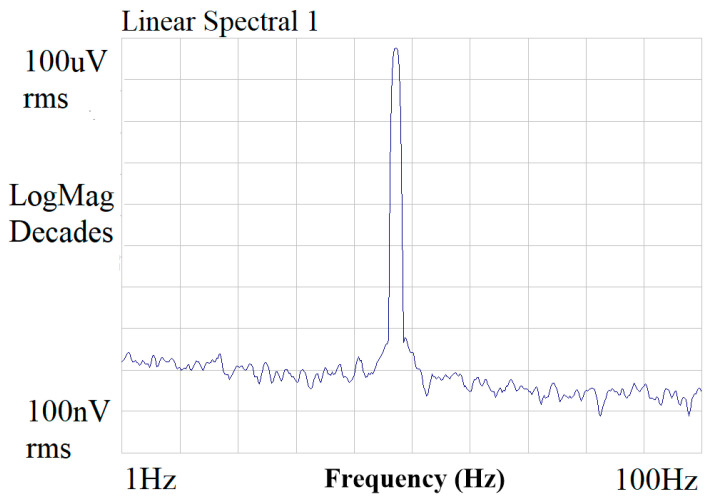
Noise test.

In our work, we use a power supply voltage of 5 V. The power consumption of the analog modulator is about 6 mW, and the power consumption of the digital filter is about 7 mW. The power consumption per block is shown in Figure 12. To validate the effectiveness of the chopper technique, we utilized a spectrum analyzer to test the circuit′s noise, and the noise results are shown in Figure 13. The 1/*f* noise corner frequency is reduced from 10 kHz to 0.7 Hz by the chopper technique.

**Figure 14:**
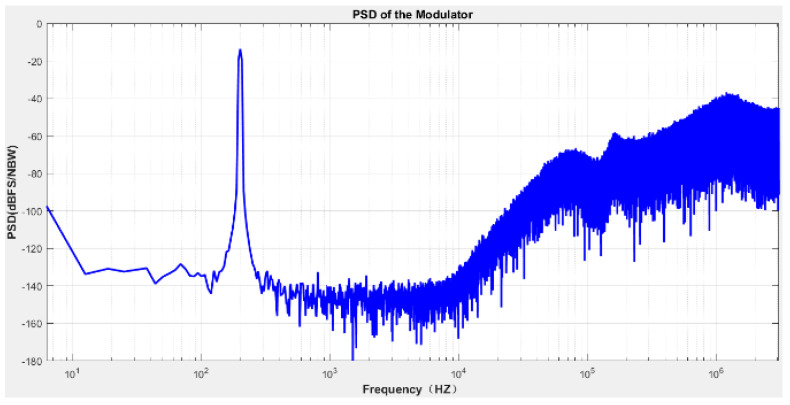
Test of the output PSD.

In order to further evaluate the performance accuracy of a sigma-delta ADC, it is necessary to analyze the spectral characteristics of the output digital signal and test the signal-to-noise ratio and harmonic distortion. When the input signal has an amplitude of 60 mV and a frequency of 200 Hz, the output spectrum is as shown in Figure 14. Figure 14 shows the output signal spectrum after 16-time quantization averaging. The maximum input signal amplitude of sigma-delta is −3 dBFs. The test results show that the noise floor is −135 dB (@1 Hz), the dynamic range is 118 dB, the bandwidth is 24 kHz, the maximum signal-to-noise ratio is 110.5 dB, and the practical bit number is 18.06 bits. We compared our work with other reported works to evaluate sigma-delta ADC performance, as shown in Table 5. Table 5 summarizes the basic test parameters of the sigma-delta ADC. Although the FOM (Figure of Merit FOM = P/BW × 10^DR/20^) of this work is smaller due to the disadvantage of the process, the comparison shows that this work achieves more excellent performance, and the circuit in this work consumes less power compared to the Ref. [29], and the Refs. [30,31] achieve a lower power consumption, but its dynamic range is lower compared to this work.

## 5. Conclusions

This work proposes a high-precision 24 bit−24 kHz sigma-delta ADC chip for Wheatstone bridge sensors. The designed circuit has the advantages of low noise, low power consumption, and high stability. The fourth-order CIFF topology sigma-delta modulator is designed and optimized at the system level. The circuit adopts a fully differential structure to reduce harmonic distortion. The first-stage CIC filter adopts a “5-stage integrator-extractor-5-stage differential” structure, which conveniently implements the function of variable extraction factor. The overall filter’s output sampling frequency and passband cut-off frequency are controlled. The chip is processed by a 0.35 μm CMOS process, and the PCB test circuit is designed and tested. The sampling frequency of the chip is 6.144 MHz, the maximum input signal amplitude is −3 dBFs, the noise floor at low frequency can be reduced by about 20 dB by using CDS technology, the dynamic range of 1 Hz is about 118 dB after using chopping technology, the signal-to-noise ratio of the modulator can reach 110.5 dB at a signal bandwidth of 24 kHz, the practical bit is about 18.05 bits, and the harmonic distortion is about −113 dB, which meets the design requirements. The overall power consumption of the circuit is 13 mW, and the output bit stream is 24 bits. 

## Figures and Tables

**Table 1:** The parameters of the Sigma-delta modulator.

	c1	c2	c3	c4	a1	a2	a3	a4	b	g
SDtoolbox	0.27	0.42	0.31	0.19	2.9	2.6	1.8	1	-	-
Optimization factor	1/3	1/2	1/4	1/5	3.2	2.8	2.4	2	1/2	1/100

**Table 2:** The capacitance values and the equivalent loads on the operational amplifiers.

	First Stage	Second Stage	Third Stage	Fourth Stage
Sampling capacitance	2.8 pF	1 pF	1 pF	0.5 pF
Integrating capacitor	8.4 pF	2 pF	4 pF	2.5 pF
Feedforward Summing Capacitor	1.6 pF	1.4 pF	1.2 pF	1 pF
Feedback Coefficient (β)	3/4	2/3	16/21	10/13

**Table 3:** The specifications of the second-stage CIC filter.

Performance Metrics	Metric Values
Input Sampling Frequency	64 kHz
Passband Cutoff Frequency	9.366 kHz
Stopband Cutoff Frequency	15 kHz
Passband Ripple	0.001 dB
Stopband Attenuation	90 dB
Order	34
Decimation Factor	2
Input/Output Bit Depth	26/24

**Table 4:** The specifications of the third-stage FIR filter.

Performance Metrics	Metric Values
Input Sampling Frequency	32 kHz
Passband Cutoff Frequency	5.876 kHz
Stopband Cutoff Frequency	7 kHz
Passband Ripple	0.005 dB
Stopband Attenuation	53 dB
Order	54
Input/Output Bit Depth	24/24

**Table 5:** Comparison of the performance of sigma-delta in this work and those reported works.

	Process	Voltage	Area	Power Consumption	Bandwidth	SNDR/DR	FOM
[29]	0.6 μm	5 V	12.16 mm^2^	50 mW	0.4 kHz	104.9/- dB	-
[30]	0.18 μm	3 V	1.22 mm^2^	5.6 mW	20 kHz	88.7/99 dB	3.14
[31]	0.18 μm	0.9 V	0.31 mm^2^	0.06 mW	10 kHz	-/70.2 dB	1.85
[32]	0.5 μm	1.8 V	0.4 mm^2^	1.7 mW	11 kHz	62/80 dB	15.4
This work	0.35 μm	5 V	9 mm^2^	13 mW	24 kHz	110.5/118 dB	7.7

## Data Availability

All data generated or analyzed during this study are included in this article.
